# Consumer Seafood Waste and the Potential of a ‘Direct-from-Frozen’ Approach to Prevention

**DOI:** 10.3390/foods10112524

**Published:** 2021-10-21

**Authors:** Roni A. Neff, David C. Love, Katie Overbey, Erin Biehl, Jonathan Deutsch, Irena Gorski-Steiner, Pete Pearson, Toriana Vigil, Catherine Turvey, Jillian P. Fry

**Affiliations:** 1 Johns Hopkins Center for a Livable Future, Bloomberg School of Public Health, Johns Hopkins University, 111 Market Place, Suite 840, Baltimore, MD 21202, USA; dlove8@jhu.edu (D.C.L.); ebiehl1@jhu.edu (E.B.); 2 Department of Environmental Health and Engineering, Johns Hopkins Bloomberg School of Public Health, Johns Hopkins University, 615 N Wolfe St, Baltimore, MD 21205, USA; koverbe1@jhu.edu (K.O.); igorski1@jhu.edu (I.G.-S.); 3 College of Nursing and Health Professions, Drexel University, 3141 Chestnut Street, Philadelphia, PA 19104, USA; st96d633@drexel.edu; 4 World Wildlife Fund, 1250 24th St NW, Washington, DC 20037, USA; pete.pearson@wwfus.org; 5 Department of Chemical and Biomolecular Engineering, Johns Hopkins Whiting School of Engineering, 3400 N Charles St, Maryland Hall 221, Baltimore, MD 21218, USA; tv4qf@virginia.edu; 6 Milken Institute School of Public Health, The George Washington University, Washington, DC 20052, USA; cturvey@gwmail.gwu.edu; 7 Department of Health Sciences, Towson University, 8000 York Road, Towson, MD 21252, USA; jfry@towson.edu

**Keywords:** food waste, seafood, fish, frozen, retail, consumer, opinion

## Abstract

Few food waste interventions focus on drivers distinct to particular food groups, such as seafood. Given suggestive evidence that seafood may be wasted at exceptionally high rates, and given its environmental, economic and nutritional value, this research provides insights into seafood-specific consumer food waste interventions. We performed three complementary sub-studies to examine consumer and retailer views regarding seafood waste and frozen seafood as well as perceptions of an intervention providing chef-created recipes to promote cooking frozen seafood without defrosting. The findings indicated an openness to a direct-from-frozen intervention among many consumers and retailers, and suggested seven potential barriers to adoption, along with ways to address them. Underlying the potential for this intervention, and more broadly contributing to addressing consumer seafood waste, the research formed the basis of a new “4 Ps” concept model to characterize the drivers of discarded seafood: proficiency, perceptions/knowledge, perishability, and planning/convenience. These factors shape waste through pathways that include behavioral protocols; taste preferences; waste-prevention efforts; and food safety concerns, precautions, and errors. This research suggested the benefit of testing a larger-scale direct-from-frozen intervention using insights from the concept model and, more broadly, the benefits of exploring approaches to food waste prevention rooted in specific food groups.

## 1. Introduction

In the U.S. and elsewhere, national leaders have committed themselves to halving consumer food waste by 2030 [1,2]. While a growing body of literature explores the drivers of food waste, in most cases all types of food are grouped together. There may be considerable benefit from linking evidence about consumers’ distinctive attitudes and behaviors toward a particular food category (such as seafood) or a food form (such as frozen), with broader evidence about factors influencing food waste, to design and refine prevention interventions.

Up to 40–47% of the U.S. seafood supply may be lost or wasted, according to a 2015 analysis by Love and colleagues [3], while globally the United Nations estimates that 35% of all harvested seafood is later lost or wasted [4], but there are few published estimates of the quantity wasted at the retail and consumer levels. In the U.S., consumers obtain the majority of their seafood from grocery stores, supermarkets, and club stores [5]. Retail waste, referred to by the industry as “shrink”, includes discards of expired seafood, products with freezer burn, damaged items, and stolen goods. Buzby and colleagues estimated U.S. retail shrink rates of 21.3% for fresh fish and 24.1% for fresh shellfish (crustaceans and mollusks) [6] while O’Donnell et al. (2021) report that, anecdotally, loss rates from seafood counters are 8–20% [7]. At the consumer level, Muth and colleagues used a supply-side approach to estimate that U.S. consumers waste 40% of fresh and frozen fish and shellfish and 17% of canned tuna and salmon. Most bottom-up consumer-level waste quantifications bundle seafood with meat. For example, in one of the most robust U.S. estimates, the Natural Resources Defense Council found 3–4% of all household food waste was fish or meat [8]. While tracking meat and fish waste together is efficient, factors such as consumers’ greater familiarity with meat may lead to different waste patterns.

Given seafood’s environmental impact (higher than many other foods, though below many terrestrial animal products), nutritional value, and often relatively high price compared to most other foods, discarding seafood has an outsized effect [9,10,11,12,13]. Seafood waste, like general food waste in higher income countries, may be especially substantial among consumers, and in retail and restaurants compared with further up the supply chain [3,14]. The embodied environmental consequences of waste at the consumer end of the supply chain include not only the resource, greenhouse gas emissions and other impacts from production, but also the effects of processing, transportation, storage and preparation. Accordingly, designing effective interventions to reduce consumer seafood waste is a priority.

While consumer seafood decision-making has many commonalities with decisions about other foods (such as prioritization of taste, price, health, convenience and habit [15,16,17,18]), distinctive features of our relationship to seafood may contribute to different waste patterns. The distinctiveness of seafood begins with the food itself, particularly its high perishability and strong odor from fish oils that can raise safety and quality concerns as well as distaste [15,17,19]. Consumers often have a relatively low level of proficiency (both skill and self-efficacy) in managing seafood compared to other foods. Additionally, they may have anxieties linked with concerns about foodborne bacteria and broader risks in the seafood supply, such as mercury content [15,19,20]. Several studies support the centrality of habit and familiarity in seafood choices, including reliance on tried-and-true purchasing and recipes [20,21,22]. Finally, frozen seafood may be preferred to fresh for its lower price, or avoided due to perceptions about lower quality.

The World Wildlife Fund and the Drexel Food Lab at Drexel University developed a seafood waste intervention that addresses many of these factors, specifically, cooking seafood “direct-from-frozen” without defrosting [7,23]. Students and faculty at the Drexel Food Lab developed a set of recipes and tested them with consumers. The intervention was premised on the idea that keeping seafood frozen for as long as possible prevents retail and consumer waste. The intervention’s implicit theory of change could be said to be rooted in the motivation–opportunity–ability framework [24,25]: shifting consumer behavior to increase the practice of cooking direct-from-frozen requires motivation (the perception that the food will meet the goals for taste, convenience, price, quality, while addressing emotional barriers, such as anxiety about taste and food safety); opportunity (availability of frozen seafood and recipes); and ability (skills, self-efficacy).

O’Donnell and colleagues studied a supermarket intervention involving the provision of these recipes, a brochure, and four weekly taste sessions at a suburban Philadelphia, PA, supermarket [7]. They found that consumers reported that the task of defrosting deterred them from purchasing of frozen seafood, and half of the 100 surveyed consumers expressed positive views about the idea of cooking directly from frozen. Prior to the taste tests, 7% said they already cooked direct-from-frozen. Afterwards, participants were asked how they would prepare the fish they sampled if they purchased it: 34% said they would cook it direct-from-frozen.

This study aimed both to add insight regarding this intervention and its potential for acceptance, and more broadly to shed light on the drivers of consumer seafood waste including by presenting a new concept model to inform further development and implementation of this and other interventions. It also contributed to the scant literature on seafood waste quantification by sharing new data on the estimated amount of seafood discarded by retailers and consumers. The study triangulated findings from three complementary explorations of consumer seafood waste, frozen seafood, and the direct-from-frozen intervention: interviews with supermarket seafood managers, consumer food diaries/surveys, and consumer focus groups.

## 2. Materials and Methods

The following section details the three types of data collection. See Materials S1 for a graphic research scheme.

### 2.1. Food Retail Interviews

We performed eight semi-structured interviews with employees at grocery stores and supermarkets in the Baltimore, MD, area in 2018. Retailers were selected using the Maryland Food System Map as a sampling frame (Maryland Food System Map Project, 2019). We randomly selected from lists of chain and independent stores, and then chose locations based on convenience. Only stores with both fresh and frozen seafood were included in the study. Sixteen eligible stores were contacted and eight participated, with four refusing and four not scheduled after repeated follow-up attempts. Interviews of approximately 20–30 min were conducted with four seafood managers or assistant managers on-site in employee break rooms or outdoor benches as selected by each interviewee for privacy and convenience. For six of the eight interviews, the conversations were audio recorded and notes were taken based on the recordings, while two interviewees preferred only written notes. The interview guide covered topics including managers’ perceptions of consumers’ shopping habits, store stocking of fresh and frozen products, marketing and profitability of fresh and frozen products, shrink, and opportunities to reduce seafood waste (Materials S2). Interviewees each received a USD 30 gift card for participation.

### 2.2. Consumer Seafood Waste Diaries and Surveys

We developed and implemented an online two-week food diary in October and November 2018. Participants were invited to take a screening survey through a post on Craigslist in Baltimore and nine other cities using a random number generator from among sites served by Craigslist in September, 2018: Cleveland and Sandusky, OH; Eugene, OR; Greensboro, NC; Hudson Valley, NY; Jackson, MS; Janesville, WI; Salina, KS; and Sierra Vista, AZ. To meet the inclusion criteria, they had to be 18 years or older, reside in the U.S., speak and write English, go grocery shopping at least once a month, cook food at home at least once a month, and eat seafood, excluding canned tuna, at least once a week. The last criterion was added to increase the number of seafood items that would be included in the diary. If participants met the inclusion criteria in the screening survey, they were directed to a pre-diary survey and contacted about participation in the two-week food diary.

The pre-diary survey (Materials S3) included 17 questions about seafood consumption attitudes and behaviors, and about demographics, while avoiding questions about discards. The survey included questions that had been used in other food waste consumer surveys including Neff et al. [26], with some adaptations to the context of seafood, as well as several original questions developed based on research goals.

The seafood discard diary (Materials S4) that we piloted here was novel because it was aimed at calculating the discard percent, not just the quantity, and because it focuses in on a single product. The tool tracked seafood items from purchase to discard using piped-in text. Participants received three initial daily questions asking if they prepared, purchased, or discarded seafood that day. If the answer was yes, they answered follow-up questions about the relevant behaviors and seafood items. The diary length varied based on responses and whether the data could be piped in from prior day responses about the item. Up to five additional questions were asked about each item, up to eight about each prepared item, and up to 10 about each discarded item. Questions included purchase location, seafood form and type, amount of product, packaging information, preparation method, and reason for discard. Data were analyzed based on individual items entered. Participants were sent a text or email reminder each day for 14 days with the diary webpage link and were able to record missed days within 48 h.

After completing the full diary, participants were directed to a post-diary survey (Materials S3) including 24 primarily closed-ended questions on diary experience, general seafood and waste behaviors, and seafood knowledge. After the post-diary survey, participants were mailed gift cards that were scaled based on diary days completed, up to a maximum of USD 50.

Pre- and post-survey results were analyzed descriptively. Diary results were analyzed through a complex process to link food items across days and were then examined descriptively.

In total, 282 people took the screener survey and 121 qualified to take the pre-diary survey. All responses were kept for analysis. We then invited the first 68 individuals to participate in the two-week diary, restricting enrollment to seven per state to support geographic diversity. Forty-three individuals participated in the two-week diary, 42 of whom took the post-diary survey.

### 2.3. Consumer Focus Groups

To understand consumer perceptions of seafood and frozen seafood in greater depth, we conducted seven 1 h focus groups (*n* = 38 total participants) in the Baltimore metropolitan area between March and June 2018. Participants were recruited via Craigslist postings in Baltimore and Annapolis. To meet the inclusion criteria they had to be 18 years or older, reside in the United States, speak and write English, go grocery shopping at least once a month, cook food at home at least once a month, and purchase seafood, excluding canned tuna, at least once a month. Questions focused on factors that affect seafood purchasing decisions, sourcing, perceptions of fresh versus frozen, frequency of purchasing frozen products, perceptions of cooking directly from frozen, and reactions to two direct-from-frozen recipes. (Materials S5). Participants submitted an anonymized demographic sheet at the end and received USD 30 gift cards for participation. Focus groups were conducted in a private room at a public library, and snacks and beverages were provided.

Recordings were transcribed by Production Transcripts and the data were analyzed using Atlas.ti. The analysis used a pragmatic, iterative approach that drew insights both directly from the data and from reflections and linkages to literature and ideas [27]. We developed code families based on research questions, and the codebook was developed iteratively to include both inductive and deductive codes. Deductive codes covered topics specifically addressed in the interview guide, while multiple inductive codes were created to cover different product attributes (Materials S6). The final codebook contained 14 code groups covering participant characteristics, actions; considerations, cost, feelings/emotions, freezing, frozen frequency of purchase (scale), frozen willingness (scale), issues, location of purchase, products, product attributes, store attributes, and waste. Within these, 99 distinct codes were created. Two researchers coded transcripts individually and then met to assess intercoder agreement and reconcile differences. For this analysis, we summarized findings from the most frequently used codes first, and then continued to additional codes until saturation. Memos were created to summarize insights for codes or groups of related codes, and key quotes illustrative of important themes were highlighted and summarized. Insights from the synthesis were checked by returning to the data and seeking counterexamples. Tables were organized based on categories that emerged iteratively from the coding and the literature, and informed the constructs in the concept model. In most cases, qualitative results were not quantified because qualitative evidence is intended to provide depth of insight and hypothesis generation rather than representative findings [27].

### 2.4. Concept Model

This study presented a new concept model informed by the analyzed data and by bodies of research on consumer seafood and food waste behavior. Given the size of the literature, we drew on syntheses of major themes rather than relying on smaller, individual studies. These synthese included a comprehensive 2020 literature review on consumer food waste from a U.S. National Academy of Sciences, Engineering, and Medicine (NASEM) panel [24] (on which the lead author of this manuscript served), four reviews on consumer seafood behavior, and a recent comprehensive seafood survey of U.S. consumer attitudes and behaviors. [15,16,17,18,20] The concept model was built iteratively, first by reviewing the current findings and mind-mapping the relevant insights about consumer seafood waste behavior. The model was then edited and shaped based on relevant insights from these two separate sources. The main categories of seafood drivers were directly linked to 11 categories of food waste drivers from the NASEM report, which can be seen in a Supplementary Table S2.

## 3. Results

We presented the results topically so that findings across the three complementary respondent samples may be considered jointly. As background, we summarized the sample and reported the amounts of seafood waste. We then provided further context for developing seafood waste interventions by characterizing reported perceptions and attitudes related to seafood waste and frozen seafood. Lastly, we presented findings regarding the idea of cooking seafood directly from frozen.

### 3.1. Sample

The eight Baltimore retail seafood managers came from four large supermarket chains, two local supermarket chains, and two independent grocery stores. Full demographics for those who participated in the 14-day food diary survey are provided in Table S1. Among the 43 diary completers, the largest age group was 30–44 (49%), 79% were female, and 52% had household incomes above USD 75,000. Seventy percent were non-Hispanic White, 11% were non-Hispanic Black, 14% were Asian, and 2% were other. Overall, these respondents were younger and more educated than the U.S. average. The focus group participants (*n* = 38) ranged in age from 21 to 69. Sixty-one percent were female, 55% were Black and 34% White, and the majority of household incomes were USD 25,000–49,999 (37%), or USD 50,000–74,999 (26%). Overall the groups had lower incomes and a higher Black population than the U.S. average.

### 3.2. Seafood Waste Quantification

#### 3.2.1. Retail Seafood Managers

The managers each reported selling 20–130 stock keeping units (SKUs, distinct products) of seafood where the frozen percentage ranged from 40 to 90%. They uniformly reported that shrink rates were lower for frozen products than for fresh. Those who tracked shrink quantitatively reported a range of 3–7% per week for fresh and zero or close to zero for frozen products (Table 1). We did not collect data on shrink rates for canned seafood. Reasons given for shrink included products not being sold in time, theft, customers leaving an item in another area of the store, damage to products, staff errors, freezer burn, and freezer malfunction. Inventory management was described by all interviewees as a critical factor for minimizing seafood shrink.

#### 3.2.2. Consumers

Survey: The average household in the study discarded an estimated 10.5 g/day of seafood over two weeks, reflecting 10.5% of the seafood purchased or 25.0% of the seafood prepared. We assessed whether some product forms were wasted more than others by comparing the relative rates purchased or prepared at home to the relative rates discarded (Table 2). Shelf-stable products (e.g., cans, foil-lined pouches) were discarded at the lowest relative rates, comprising 20% of purchased products but only 12% discarded. By contrast, seafood purchased in prepared or cooked form comprised 12% of purchases and 24% of discarded items. Fresh and frozen products were both discarded in similar percentages to the amount purchased. In the post-diary surveys, the median typical reported time between purchase and preparation was 3 days for fresh, 20 for frozen, and 60 for canned.

The experience of tracking seafood waste led participants to view their waste as relatively low. Prior to the diaries, 32% said they wasted less seafood than the average American and 45% said about the same, while after the diaries these percentages shifted to 62 and 33%.

**Figure 1:**
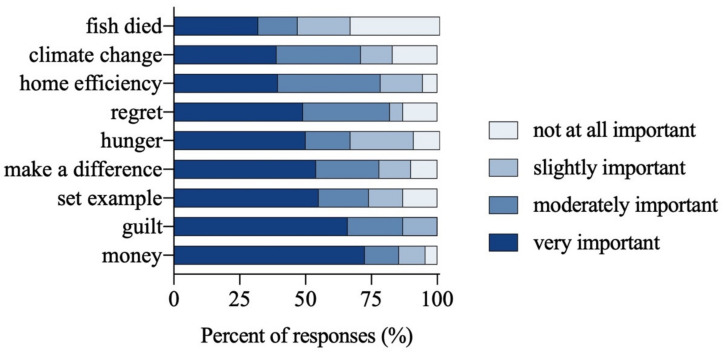
Motivations for reducing seafood discards from a post-seafood diary exit survey (*n* = 42 participants).

The post-diary surveys provided insights into factors affecting seafood discards. The most common reasons were related to perishability and planning: smell for both fresh and frozen (37%), followed by the date label for fresh items (21%), and leftovers perceived to have gone bad for fresh items (19%). Respondents said that household members “often” (69%) or “sometimes” (26%) agreed about when it was time to discard seafood. Respondents were also asked to consider motivating factors to reduce seafood discards (Figure 1). Feeling guilty about waste in general (87%), saving money (85%), regret about time spent (82%), and home efficiency (79%) were the most frequently selected motivating factors, while altruistic reasons such as climate change (71%) and recognition that fish had died were less motivational (46%). From a list of 8 items, we asked participants what information or tools if any they would need to reduce the amount of seafood their households discarded. The top responses were related to food literacy/food safety: how to tell when seafood may have become unsafe (50%), how long seafood can be left unrefrigerated (48%), and how to buy and cook seafood that is frozen (45%).

Focus group: Many participants described themselves as wasting no seafood, while a portion did say that they discarded seafood at times with the word “rare” frequently used as a descriptor. There was significant anti-waste discourse within the focus groups and a number of participants expressed pride in not wasting. Many rooted their negative attitudes toward waste in their families’ or grandparents’ values. Participants also frequently characterized their attitudes toward waste as embedded in their personal or household identities.

The most common reported factors leading to discarding seafood focused on planning and suggested unavoidability: waiting too long to cook or eat it, changing plans, or forgetting about leftovers in the refrigerator or freezer. Others were perishability (odor) and taste (dislike of leftovers). Some described personal protocols about when to discard seafood, and others described proficiency issues; that is, incidents where they were not sure if the food was still okay to eat, suggesting that discards may have been more common than was admitted or recalled during the focus groups. Table 3 presents illustrative quotes about reasons for discards, organized by seafood waste drivers (Table 3).

### 3.3. Attitudes Regarding Frozen Seafood

Given the focus on an intervention using frozen seafood, we wanted to understand attitudes and behaviors toward frozen seafood among retailers and among consumers who typically do—and do not—purchase frozen. Purchasing shifts among the latter may be particularly meaningful for reducing waste.

#### 3.3.1. Retail Seafood Managers

When asked what motivated consumers to purchase frozen seafood, retail staff most often mentioned price. For non-frozen seafood, retailers thought consumers valued freshness above all. Regarding opportunities for switching customers from fresh to frozen, one mentioned protocol/habit because consumers commonly buy what their parents bought. Another said, “if people were convinced that frozen was more sustainable [due to reduced waste] and they could save money, they would probably buy it more often.”

When asked about the impact of frozen seafood on their businesses, most store managers said frozen was more profitable than fresh because it has a longer shelf life, requires less labor, and creates less waste. One store indicated that they sometimes made no profit or took a loss on fresh seafood. A few stores in higher-income areas indicated that fresh was more profitable than frozen. These statements do not necessarily conflict; retailers sell to different segments of a market at differing price points.

Fresh and frozen seafood marketing practices varied among the stores. Most said there was little distinction between fresh and frozen in decisions about which products to advertise in circulars, but one said frozen items were less commonly featured. In two chain stores, sales staff said they do not control when seafood is advertised, while staff at independent stores had more control over marketing and setting discount prices. These findings suggested that marketing and promotion for frozen seafood may require different levels of involvement and buy-in from corporate leadership compared to fresh seafood.

#### 3.3.2. Consumers

**Figure 2:**
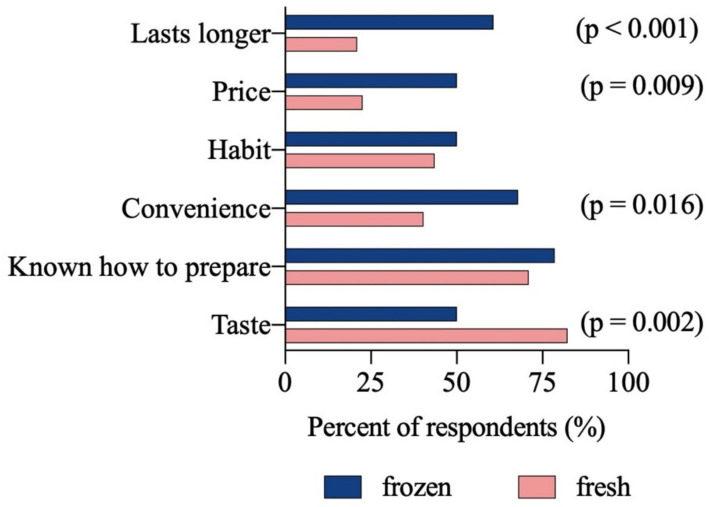
Reasons for purchasing fresh and frozen seafood from a survey of participants eligible to be enrolled in the seafood diary (*n* = 121 participants).

Survey: Consumers have different reasons for purchasing fresh and frozen products, which partially aligns with the retailers’ perceptions of consumers. In the pre-diary survey, participants who bought mostly frozen reported that proficiency (79%), convenience (68%), and perishability (61%) were the most important considerations (Figure 2). Buyers of fresh seafood made purchasing decisions mostly based on taste (82%) and familiarity with preparation (71%) (Figure 2). Eighty-three percent of diary participants were aware that, at least sometimes, seafood labeled as fresh had been previously frozen.

Focus group: Participants also distinguished between fresh and frozen preference (Table 4). At first, most said they preferred fresh over frozen seafood often because they perceived it as higher quality or because of the confidence they felt when they could see and smell the quality more directly when shopping. Many expressed concern and distrust related to how long the seafood may have been frozen, and a negative perception of the preservatives they understood to be used in freezing. Food safety concerns were widespread, and often connected to distrust of processors or others in the food system. Regarding planning, those who preferred to buy fresh seafood commonly indicated they would buy it with specific meals in mind, whereas frozen was often more valued by those who were uncertain when they would cook it. Both fresh and frozen were referred to as more convenient, but for different reasons: fresh because consumers did not need to take the time to defrost it, and frozen because it was available in their freezers, thereby avoiding the need for an extra shopping trip. Several mentioned that frozen products were more available in areas of the city with fewer supermarkets. Several mentioned the benefit of frozen packages containing individually wrapped items, which meant they could take some out and save the rest for later. Most participants had not been aware that seafood sold as fresh was often previously frozen.

### 3.4. Views on Cooking ‘Direct-from-Frozen’

Lastly, we explored reactions to the idea of cooking seafood directly from frozen both in general, and in context of specific chef-developed recipes.

#### 3.4.1. Retail Seafood Managers

Most retail seafood managers had not previously heard of promoting cooking ‘direct-from-frozen’ seafood, and about half were skeptical of consumer adoption. Two interviewees reported that consumers tend to be set in their ways. One said, “You don’t get many converts between fresh and frozen,” while another stated that consumers buy the same thing every time they shop. Other respondents felt the opposite and thought consumers might be open to it based on convenience. Several said the approach might appeal to parents with children, young adults, and those who prioritized convenience and speed (“grab and go”). None said they would avoid promoting it (e.g., via conversations with customers or in-store marketing).

#### 3.4.2. Consumers

Survey: The post-diary survey stated that some chefs are proposing the idea of buying frozen seafood and cooking it without defrosting it. Most respondents (60%) had never tried this. Of those who had previously done so, 65% said they had not noticed a difference in flavor from seafood that had been defrosted. Of those who had never done so, 72% were willing to try it for a variety of reasons (trying new recipes, saving money or time, being open to trying it once, or avoiding waste.) Those who did not want to try cooking direct-from-frozen seafood were primarily concerned about flavor, food safety, or that their dish would not cook properly. Respondents were then asked to review a recipe from the intervention (e.g., for Roasted Tilapia with Creamy Tartar Sauce (Materials S7). Afterwards, 45% said they would try preparing it, while 24% said they would not.

Focus groups: Most participants had not previously cooked direct-from-frozen seafood and there was initially low receptivity to the idea. Participants were most frequently concerned about whether it would cook appropriately and whether it would be safe (Table 5). We then presented two recipes: Butter Poached Garlic Shrimp and Roasted Tilapia Sheet Tray Dinner (Materials S8). These concrete, chef-created examples shifted the conversation toward positive views of direct-from-frozen cooking (Table 6). They reported that learning that much fresh seafood had been previously frozen also contributed to their openness. Participants said the recipes looked healthy, tasty, easy, or they would be interested in trying something new if they had a recipe to follow. Some, however, were still concerned about it cooking evenly or poor flavor.

## 4. Discussion

This study presented triangulated data from retailers, consumer diary survey participants, and focus group participants both to contribute to the theory about consumer seafood waste via a new concept model, and, to explore potential openness to the direct-from-frozen approach to prevention based on those insights. The findings supported the insight that it can be beneficial to elucidate food waste drivers by food type. Distinctive features of seafood, particularly perishability, consumers’ level of proficiency and their perceptions, and the related anxieties and reliance on habit and precedent, lead to a waste profile that differs in some ways from those of other foods. These features can inform distinctive approaches to prevention, such as cooking seafood directly from frozen. The research found openness to the intervention among consumers and retailers, particularly when chef-created recipes were shared. The seven identified barriers to adoption may be addressed via the study’s insights about consumer attitudes and behaviors toward seafood, seafood waste, and frozen seafood.

### 4.1. Concept Model

**Figure 3:**
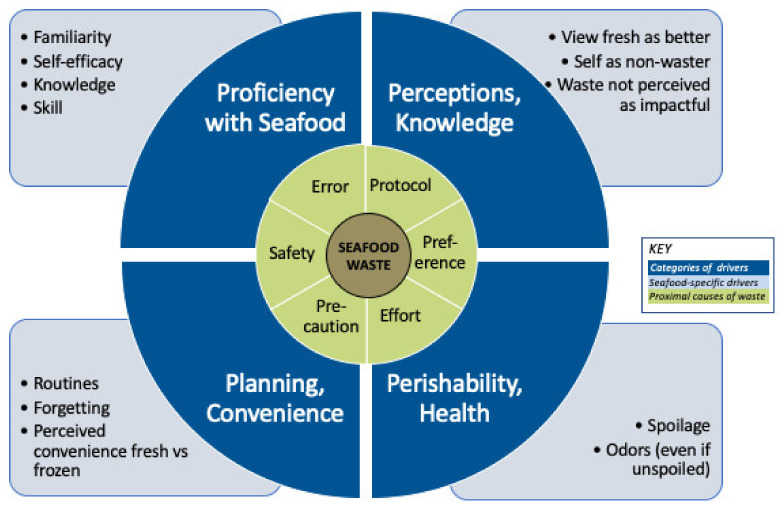
Seafood Waste Drivers Concept Model.

This research supports theory-building regarding seafood waste drivers and the ways they may affect both waste and receptivity to the direct-from-frozen intervention. We found that the reasons for seafood shrink and consumer discards were fairly similar across the three samples, and that the findings link together insights drawn from the separate literatures on seafood consumer behaviors and wasted food. Based on these findings and the literature, we created a new concept model summarizing key drivers of consumer seafood waste and the proximal causes through which they operate (Figure 3).

Drivers: The model highlighted four categories of drivers that are generally not unique to seafood; however, most play a stronger or distinctive role in seafood waste compared to food waste generally, due to seafood’s material properties and the ways in which consumers commonly relate to it. (Table S2 shows how our categories align with the 11 categories of food waste drivers identified by the NASEM report [24])

Proficiency/familiarity: Seafood is less frequently eaten in the U.S. and many other countries compared to terrestrial meats [4,5]. Consumers often have relatively low familiarity, self-efficacy, knowledge and skill regarding seafood selection, handling and risk assessment [15,17,19,20,28] which may lead to unnecessary discards via errors or caution. Many compensate for low proficiency and related anxieties by limiting selections to familiar products or holding relatively rigid “protocols” for seafood selection, handling, recipes and storage [15,16,17,18]. Even when such protocols guide consumers toward practices that reduce waste of a costly product, they still can inhibit openness to approaches that may be even more effective at preventing waste, such as using frozen seafood. Our research also found that a portion of consumers were eager to build proficiency and introduce novelty, thereby providing an important opening for seafood waste prevention.

Perceptions and knowledge: This study echoed the findings in consumer seafood research regarding common seafood-related perceptions [15,16,17,20]; here, we considered the ways these views may influence waste. Perceptions about the relative quality of fresh vs. frozen seafood, and the lack of knowledge that much “fresh” seafood was once frozen, can shape willingness to accept frozen seafood. Consumer perceptions may also be affected by widespread marketing of the concept of “freshness” as a marker for healthfulness and quality [29], while the perceived use of preservatives and other additives in frozen seafood may contribute to disgust or anxiety reactions. The level of interest in preventing waste may be low among those who perceive themselves as wasting little, and those with little knowledge of the environmental impacts of seafood production [24,26]. As is common in food waste surveys [26], the consumers in this study placed a low priority on environmental reasons for waste avoidance, yet frequently expressed disdain for food waste, and felt they wasted little overall, despite the diary estimates summarized below.

Perishability/Health: One of the top causes of consumer discards is concern about food safety and quality. Fresh seafood spoils rapidly, and seafood can cause foodborne illnesses (though raw products that will be cooked do not need to be discarded on a precautionary basis based on the date label alone [30]). Additionally, strong odors from fish oil may lead to discards via anxiety or distaste even while food remains safe [15,17,19,20]. Maintaining seafood in a frozen state generally avoids these perishability threats. In addition to food safety, we include nutrition under the “health” category. While we did not find nutrition to be a waste driver, it is one of the top drivers of seafood purchasing. Our research lends support to the idea that aligning waste prevention with consumer health goals and perceptions may increases their appeal.

Planning/convenience: We found that, as with food waste generally, one cause of seafood waste is poor planning [31]: consumers are sometimes overly optimistic about having the time to cook it before it spoils. Planning is particularly important for seafood given the short window for consumption. A direct-from-frozen approach could be particularly helpful because it would allow consumers to leave food frozen until needed. As with most foods, convenience is a top driver of decision-making for seafood [15,16,17,18,20]. Consumers in this study had mixed perceptions about whether fresh or frozen seafood was more convenient. If defrosting were no longer a barrier, as in direct-from-frozen preparation, the balance might shift toward frozen. Additional convenience benefits of frozen mentioned by participants include individually packaged frozen items and meal packages.

Proximal Causes: The model highlights six key proximal causes of seafood discards. Protocols: following a relatively rigid protocol or tradition in seafood purchasing/management may increase waste via unnecessarily precautionary discards or a limited openness to trying new approaches such as direct-from-frozen preparation. Preferences: individual and family taste preferences may lead to discards via high sensitivity to odors, rejection of leftovers, or an unwillingness to try new approaches that could prevent waste. Effort: this category includes discards due to carelessness and having a low standard about when to keep or discard. Safety: While seafood should certainly be discarded when it may be unsafe, such discards are still often preventable by eating it before it spoils or keeping it frozen. Caution: Precautionary approaches to discards are appropriate for food safety; however, too much caution can lead to unnecessary discards. Error: In contrast to precaution, this category focuses on cases where the person erroneously believes seafood should be discarded; it also includes errors in planning, storage, and cooking.

### 4.2. Direct-from-Frozen Intervention

This research finds further support for the direct-from-frozen intervention first explored in O’Donnell et al. [7] based on retailer and consumer reactions. Retailer and consumer findings suggested that consumers who already use frozen seafood, are interested in convenience, and want to try new recipes may be more open to the idea. Some retailers were skeptical that consumers would shift established practices but were not opposed to promoting the direct-from-frozen approach. In many cases they indicated that frozen seafood was more profitable, due to higher margins, less shrink, and less labor than a staffed fresh seafood counter.

The research suggested seven potential barriers to adoption and ways that these may be addressed using insights from the research. We organized these with reference to the Motivation–Opportunity–Ability framework [24,25], in which all three must be present to support a change in behavior.

Motivation: Five of the seven identified barriers to adoption were motivational factors linked with perceptions: (1) palatability to self and family, (2) quality perceptions of frozen seafood, (3) food safety concerns about direct-from-frozen preparation, (4) a view that a waste reduction strategy was not needed because seafood waste was very low; and (5) distrust of frozen seafood due to concerns about preservatives or false labeling.

Opportunity: No barriers were identified, as the opportunity to create the behavior had already been addressed via the recipes within the intervention and the widespread availability of frozen seafood.

Ability: Two proficiency-related barriers were also identified: (6) low familiarity with the idea, and (7) frequent adherence to protocols about seafood purchasing and preparation.

An education and marketing campaign focused on direct-from-frozen seafood cooking may be the most effective way to jointly address these barriers. Our research suggested that the palatability, quality and safety concerns in barriers (1–3) may be substantially addressed through the appeal of the recipes and by emphasizing that they were created and tested by chefs. Given the challenge of (4) convincing consumers that their seafood waste is significant may be more productive for highlighting additional benefits such as price, convenience, taste and novelty. The campaign can directly address (5) misperceptions, but messaging will need to go beyond providing information, to addressing underlying emotional factors such as anxiety/trust; and desires: to be a good provider, make a great meal, get one’s money’s worth, reduce household labor, avoid waste, and save money). The campaign will build (6) familiarity and comfort with the idea, creating (7) openness and a sense of safety, normativity or excitement in trying the recipes. In conjunction with the campaign, developing materials for retailers and convenience products such as frozen meal kits or grab-and-go products would also improve opportunity, along with a go-to location for recipes including those from the Drexel Food Lab and the Alaska Seafood Marketing Institute’s “Cook it Frozen!” campaign [32].

### 4.3. Seafood Waste Estimates

Lastly, the findings added to the limited literature on the quantities of discarded seafood, confirming that despite consumer perceptions of very low waste, diary participants reported discarding an estimated 10.5% of the purchased or 25.0% of the prepared seafood, which was in the range of previous findings [3,33]. The estimated fresh seafood shrink in retail (3–7%) was well under that estimated by Buzby et al. (21.3% fresh fish, 24.1% fresh shellfish), and also below the anecdotal estimate of 8–20% from O’Donnell [6,7].

### 4.4. Strengths and Limitations

The study has several limitations. This research is exploratory with relatively small and convenience-based samples. The focus group participants were relatively low-income and proportionately more African-American than the general U.S. population, possibly due to the Baltimore location. By contrast, survey and diary participants came from higher socioeconomic status backgrounds than the U.S. average (at least partly due to high seafood consumption as a screening criterion), and were disproportionately female, among other demographic distinctions. It is not clear how applicable the findings may be outside a U.S. context. A limitation of the interviews and focus groups is that respondents may have partly shifted their tones or answers due to concern about social desirability, while the survey and diary research may be limited due to recall and aspirational biases. Diary research is a far more effective way to learn about quantities discarded than surveys, however, they did not continue long enough to study all discards of frozen seafood, which may be especially likely to occur *en masse* in freezer cleanouts. Nevertheless, the mixed methodology allowed us to collect and contrast rich qualitative and quantitative data and gain insight into both retailer and consumer perceptions, thus providing a broader perspective on intervention feasibility and approaches to implementation.

Further research is needed with larger samples and different approaches to explore consumer attitudes and behaviors related to seafood waste, including testing the proposed concept model, as well as further research on frozen seafood and cooking direct-from-frozen as a waste prevention strategy. Intervention evaluations should examine pre- and post-consumer impacts, acceptance and frequency of direct-from-frozen cooking over time among those exposed to the intervention, as well as the exploration of feedbacks and spillovers onto other behaviors. It will be important to assess the extent to which seafood languishes in freezers and is eventually discarded. Effective evaluation of a frozen seafood intervention will require further methodological development in the food waste space, to better assess freezer discards over time in a cost effective and representative way.

## 5. Conclusions

Seafood waste has a significant environmental impact and represents the loss of a high value, nutritious food. This research suggests a distinctive profile of seafood waste drivers, in categories of consumer perceptions/knowledge, perishability, planning/convenience, and lack of proficiency with seafood. Many consumers perceived fresh seafood to be higher quality and healthier than frozen, while in some cases, frozen seafood raised food safety concerns. The insights about attitudes and behaviors toward seafood waste and frozen seafood contribute to development and refinement of waste prevention intervention approaches, including efforts to increase knowledge of and comfort with seafood generally. The research provided support for the idea of promoting cooking seafood direct-from-frozen without defrosting. Larger scale implementation and testing is warranted. Because adoption may hinge on confidence regarding taste, quality and safety, the provision of chef-tested recipes is beneficial, as is highlighting convenience, price, opportunity for novelty, and the fact that some seafood sold as fresh was previously frozen. This research supports the insight that food-specific strategies can provide a novel and valuable approach to food waste reduction.

## Figures and Tables

**Table 1:** Seafood shrink rates in Baltimore, Maryland area grocery stores and supermarkets by product form.

Store Type	Geography, Store Outlets	SKUs ^a^			Shrink Rates	
		N	Fresh	Frozen	Fresh	Frozen
supermarket	national, chain	130	42%	58%	25 items or USD 300/wk	negligible
supermarket	regional, chain	100	10%	90%	5%	n/a
grocery store	local, chain	100	50%	50%	5%	“if something goes wrong like somebody leaves a frozen fish in the bread section or a freezer breaks down.”
specialty supermarket	national, chain	70	35%	65%	7%	0%
supermarket	regional, chain	50	40%	60%	3%	“not as often as fresh; only loss is from freezer burn”
grocery store	single store	50	50%	50%	unknown	unknown
supermarket	regional, chain	25	60%	40%	“I usually don’t have too much because I order little amounts”	“I really can’t tell”
budget supermarket	regional, chain	20	50%	50%	a few items a week	“next to none”

^a^ SKU, stock keeping unit; does not include canned seafood.

**Table 2:** Portion of seafood items purchased, prepared, and discarded by product form during the two-week food diary ^a^.

Seafood Product Form	Purchased at Retail (*n* = 209)	Prepared at Home ^b^ (*n* = 195)	Discarded (*n* = 59)
Fresh	36%	36%	36%
Frozen	23%	31%	24%
Shelf-stable	20%	21%	12%
Prepared/cooked	12%	6%	24%
Other	8%	6%	5%
Total	100%	100%	100%
Total (g/d/hh)	99.9 +/− 84.4	42.2 +/− 51.88	10.5 +/− 23.5

^a^ Based on a 2-week food diary with *n* = 43 respondents and their households. ^b^ Items prepared at home may have been purchased before the study period.

**Table 3:** Reasons for discarding seafood and how discards are prevented. Focus group illustrative quotes.

Driver Category [24]	Reasons for Discards	How Discards Are Prevented
Proficiency	“Then sometimes I’m thinking the second day, ‘Should I put it in the freezer?’ and then it’s like, ‘Is it too late to freeze?‘ and then I’ve frozen some that I kept out and then I never used it… because every time I would take it out, I’d say, ‘I don’t think this is that good, ‘ and I end up throwing it away.”	
Planning	“But then in the summer … there’s so many other things we’re doing, we may defrost this fresh fish and we wind up having hotdogs on the grill … and pizza or whatever, two days has passed and then I start feeling bad.”	“I’m sorry but I came from a background where you don’t throw no food away, it’s hard to come by so I make sure to plan my meals so that I don’t have nothing to waste. Or before I throw it away I’ll put it in a container and give it to a homeless guy coming down the street.”
Price		“If I buy it fresh for what I paid for it, there is no way in the world it’s going in the trash. I bought a pint of crab cakes and I made four, a pound, and I ate all four of them because I refused to put them in the trash”
Protocol	“But it’s the third day, it’s got to go.”	
Taste	“I don’t think it tastes good the next day after you cooked it, it just doesn’t have any—the refrigerator eats up sort of that flavor for me.”	

**Table 4:** Reasons for choosing fresh vs. frozen, when purchasing seafood. Focus group, illustrative quotes.

Category	Reason for Choosing Fresh	Reason for Choosing Frozen
Perishability/Freshness	“Frozen, it could be in the freezer for months and months and months”	“At least if it’s frozen it’s got an expiration date on it, right. Whereas if it’s been sitting on ice it could very well be questionable as to how long it’s been sitting.”
Perceptions/Knowledge	“I just don’t like frozen because I think… it has too many preservatives, and it’s just processed, has too many chemicals in it.”	“Sometimes I feel like having fresh fish laying around in my refrigerator would open me up to more problems with food contamination.”
Planning/Convenience	“Frozen you have to give it time to thaw out and all that. So definitely, fresh is more convenient.”	“More for the convenience of having it in the freezer and the individually wrapped is good because … you can save the rest”
Price	“If I eat seafood…it’s like a luxury food. So therefore, I want the best.”	“Like, if it’s frozen but it‘s cheap, I’d probably buy it.”
Nutrition	“I know the fresh is healthier…. Because you don’t know how long that’s been sitting there in the freezer.”	“Over the years, I’ve tried to eat more fish, so it’s just easier and cheaper for me to have fish in the freezer at all times.”
Proficiency	“Seafood, I have to admit, I’m a little fussier about because I just think things can go wrong and I don’t feel that way about chicken or beef or whatever.”	
Taste	“Definitely fresh because frozen fish, I mean some can be okay but most of the time it loses that fish taste that you love.”	

**Table 5:** Attitudes toward cooking direct-from-frozen seafood before the introduction of recipes. Focus group, illustrative quotes.

	Negative	Positive
Protocol/Novelty	“I tend to follow a lot of things that I saw my grandmother and my mother--… and I have never seen them take anything frozen and just cook it.”	“Well, I love watching cooking shows, so if I saw someone on a cooking show, like, go through these steps and I was convinced eough by watching them that it would work, I would try it.”
Protocol/Already do it	“9 times out of 10 when I’m cooking, it’s from a recipe, and the recipe either says to thaw it or do it fresh.“	“I mean, it’s like defrosting in the microwave I mean everybody practically does that…so you just defrost it in the pan which isn’t as…hard as it seems; it’s not as crazy as it seems either.”
Anxiety/food safety	“I just would just be really concerned with fully cooking it through”	
Prevent waste: Perishability, Planning	“I do think if you bought frozen and then it thaws, I do feel like it would go bad faster than when you have fresh.”	“I would definitely throw away less… I wouldn’t have to make as much, because I know I could just take it out, heat it up and eat it.”

**Table 6:** Attitudes toward cooking direct-from-frozen seafood after the introduction of recipes. Focus group, illustrative quotes.

	Negative	Positive
Convenience	“Because there‘s too many steps and too many ingredients.”	“So, this right here, the time that it takes ––30 to 45 min from frozen which I have in the freezer––So this is amazing; this is helpful.”
Protocol/Novelty	“I wouldn’t care if it took five minutes; my food‘s got to be thawed out first.”	“I think that if the knowledge was out there, that it’s pretty much all frozen in the first place, I think more people would buy more frozen fish. I don’t think it would matter as much anymore. I’d be willing to try it all. I live a little bit on the edge and I love trying new things, so I’d be all for it.”
Taste	“I can just imagine the extra fishy, fishy taste from the stuff from it not being thawed out or rinsed off.”	“Because it looks very healthy and it’s everything on there that my son can eat.”

## Data Availability

The data presented in this study are available on request from the corresponding author. The data are not publicly available due to privacy.

## Supplementary Materials

The following are available online at https://www.mdpi.com/article/10.3390/foods10112524/s1, Materials S1: Research Scheme, Materials S2: Retail Interview Guide, Materials S3: Consumer Seafood Diary Questionnaire, Materials S4: Consumer Pre- and Post-Diary Survey, Materials S5: Consumer Focus Group Guide, Materials S6: Qualitative Codebook, Materials S7: Recipe used in survey, Materials S8: Recipes used in focus group, Table S1: Demographics for Diary Completers and Full Consumer Survey Sample. Table S2: Relationship between “4 P’s” categories of seafood waste drivers and 11 categories of overall food waste drivers identified by National Academies of Sciences & Medicine Committee on a Systems Approach to Reducing Consumer Food Waste*.

## References

[1] US EPA United States 2030. Food Loss and Waste Reduction Goal. https://www.epa.gov/sustainable-management-food/united-states-2030-food-loss-and-waste-reduction-goal.

[2] 12.3.1 Global Food Losses. Sustainable Development Goals. Food and Agriculture Organization of the United Nations. http://www.fao.org/sustainable-development-goals/indicators/1231/en/.

[3] Love D.C., Fry J.P., Milli M.C., Neff R.A. (2015). Wasted Seafood in the United States: Quantifying Loss from Production to Consumption and Moving toward Solutions. Glob. Environ. Chang..

[4] FAO (2020). The State of World Fisheries and Aquaculture 2020: Sustainability in Action.

[5] Love D.C., Asche F., Conrad Z., Young R., Harding J., Nussbaumer E.M., Thorne-Lyman A.L., Neff R. (2020). Food Sources and Expenditures for Seafood in the United States. Nutrients.

[6] Buzby J.C., Bentley J.T., Padera B., Campuzano J., Ammon C., Jean C., Buzby J.T., Bentley B.P., Campuzano J., Ammon C. (2016). Updated Supermarket Shrink Estimates for Fresh Foods and Their Implications for ERS Loss-Adjusted Food Availability Data.

[7] O’Donnell T., Katz S.H., Romey A., Fulton B., Croskey L., Pearson P., Deutsch J. (2021). Retail Seafood Waste Prevention: Reducing Retail and Consumer Fresh-Fish Waste by Cooking Directly from Frozen. Food Nutr. Sci..

[8] Hoover D., Moreno L. (2017). Estimating Quantities and Types of Food Waste at the City Level.

[9] Kim B.F., Santo R.E., Scatterday A.P., Fry J.P., Synk C.M., Cebron S.R., Mekonnen M.M., Hoekstra A.Y., de Pee S., Bloem M.W. (2020). Country-Specific Dietary Shifts to Mitigate Climate and Water Crises. Glob. Environ. Chang..

[10] Troell M., Jonell M., Crona B. (2019). Scoping Report: The Role of Seafood in Sustainable and Healthy Diets.

[11] Conrad Z. (2020). Daily Cost of Consumer Food Wasted, Inedible, and Consumed in the United States, 2001–2016. Nutr. J..

[12] Clark M.A., Springmann M., Hill J., Tilman D. (2019). Multiple Health and Environmental Impacts of Foods. Proc. Natl. Acad. Sci. USA.

[13] Poore J., Nemecek T. (2018). Reducing Food’s Environmental Impacts through Producers and Consumers. Science.

[14] Commission for Environmental Cooperation (2017). Characterization and Management of Food Loss and Waste in North America.

[15] Carlucci D., Nocella G., De Devitiis B., Viscecchia R., Bimbo F., Nardone G. (2015). Consumer Purchasing Behaviour towards Fish and Seafood Products. Patterns and Insights from a Sample of International Studies. Appetite.

[16] Christenson J.K., O’Kane G.M., Farmery A.K., McManus A. (2017). The Barriers and Drivers of Seafood Consumption in Australia: A Narrative Literature Review. Int. J. Consum. Stud..

[17] Stein R., Markenson S. (2019). The Power of Seafood 2019.

[18] Lamy J., Szejda K. (2020). Literature Review: Consumer Preferences for Seafood and Applications to Plant-Based and Cultivated Seafood.

[19] Carstairs S.A., Marais D., Craig L.C.A., Kiezebrink K. (2017). How Important Are the Influencing Factors to the Decision on Whether to Provide Seafood in Infant and Young Child Feeding?. Appetite.

[20] Govzman S., Looby S., Wang X., Butler F., Gibney E.R., Timon C.M. (2021). A Systematic Review of the Determinants of Seafood Consumption. Br. J. Nutr..

[21] Birch D., Lawley M. (2014). The Role of Habit, Childhood Consumption, Familiarity, and Attitudes Across Seafood Consumption Segments in Australia. J. Food Prod. Mark..

[22] Birch D., Memery J. (2020). Exploring the Influence of Family on Adolescents’ Seafood Consumption Choices. Int. J. Consum. Stud..

[23] Deutsch J., Zeitz A., Croskey L., Fulton B., Trout R., Pearson P. Eliminating Waste from the Seafood Supply Chain: The Benefits and Challenges of “Frozen”. https://drexel.edu/~/media/Files/cnhp/Faculty%20Pub%20PDF/WWF%20%20DREXEL%20Seafood%20White%20Paper%20v5.ashx?la=en.

[24] Schneeman B.O., Oria M., Committee on a Systems Approach to Reducing Consumer Food Waste, Board on Environmental Change and Society, Food and Nutrition Board, Division of Behavioral and Social Sciences and Education, Health and Medicine Division, National Academies of Sciences, Engineering, and Medicine (2020). A National Strategy to Reduce Food Waste at the Consumer Level.

[25] Van Geffen L.E.J., van Herpen E., van Trijp J.C.M. (2016). Causes & Determinants of Consumers Food Waste, REFRESH.

[26] Neff R.A., Spiker M.L., Truant P.L. (2015). Wasted Food: U.S. Consumers’ Reported Awareness, Attitudes, and Behaviors. PLoS ONE.

[27] Tracy S.J. (2013). Qualitative Research Methods: Collecting Evidence, Crafting Analysis, Communicating Impact.

[28] Aschemann-Witzel J., De Hooge I., Amani P., Bech-Larsen T., Oostindjer M. (2015). Consumer-Related Food Waste: Causes and Potential for Action. Sustainability.

[29] Freidberg S. (2010). Fresh: A Perishable History.

[30] ReFED (2017). ReFED Date Labeling Standardization Tool.

[31] Block L.G., Keller P.A., Vallen B., Williamson S., Birau M.M., Grinstein A., Haws K.L., LaBarge M.C., Lamberton C., Moore E.S. (2016). The Squander Sequence: Understanding Food Waste at Each Stage of the Consumer Decision-Making Process. J. Public Policy Mark..

[32] ASMI Cook It Frozen. https://www.wildalaskaseafood.com/slideshow/cook-it-frozen/.

[33] Muth M.K. (2011). Consumer-Level Food Loss Estimates and Their Use in the ERS Loss-Adjusted Food Availability Data.

