# Supplementary material for: Consumer Seafood Waste and the Potential of a ‘Direct-from-Frozen’ Approach to Prevention
Source: Foods. 2021 Oct 21;10(11):2524. doi: 10.3390/foods10112524 (PMC8618751; doi:10.3390/foods10112524)
Supplement: Supplementary file 1 [file foods-10-02524-s001.zip › foods-1403296-supplementary.pdf]

Supplemental Materials

Materials S1: Research scheme 2

Materials S2: Retail interview guide.. 2

Materials S3: Consumer pre- and post-diary survey.. 4

Materials S4: Consumer seafood diary questionnaire 18

Materials S5: Consumer focus group guide.. 21

Materials S6: Qualitative Codebook.. 22

Materials S7: Recipe used in survey (developed by Drexel Food Lab) 29

Materials S8: Recipes used in focus group (developed by Drexel Food Lab).. 29

Table S1: Demographics for diary completers and full consumer survey sample 30

Table S2: Relationship between "4 P's" categories of seafood waste drivers and 11 categories of overall food waste drivers identified by National Academies of Sciences & Medicine Committee on a Systems Approach to Reducing Consumer Food Waste*.. 31

Materials S1: Research scheme

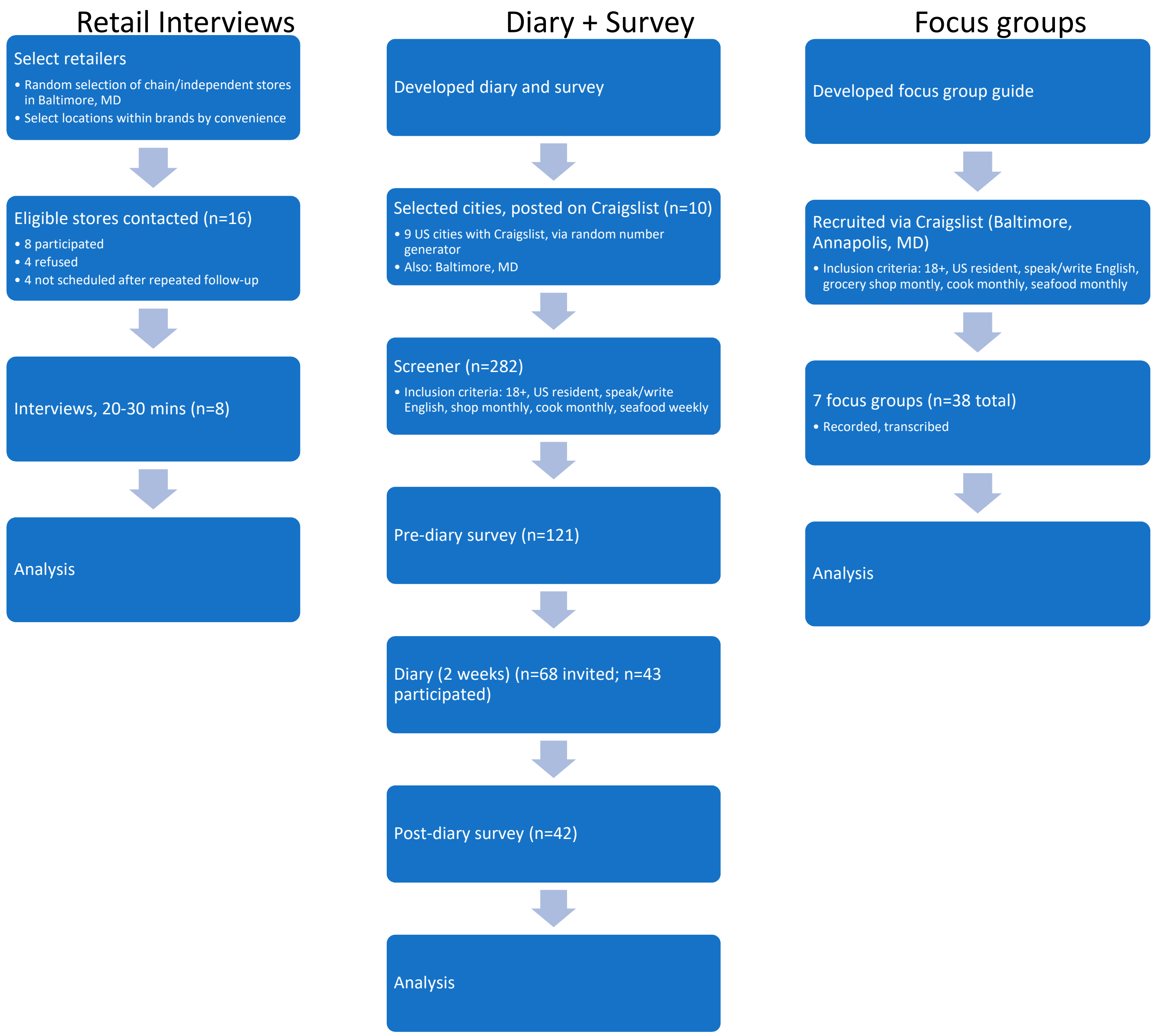

Materials S2: Retail interview guide

For our questions about seafood in your store, we are focused on unprocessed or minimally processed products, like fillets.

Background

1. What is your title at the store/company, and what are your main work responsibilities?

2. How long have you been in this position?

3. Did you work on purchasing/selling seafood before your current position?

a. (If previous experience) How long have you worked in seafood sales, and how have your

responsibilities changed over time?

4. What departments are responsible for overseeing the fresh and frozen seafood sections? Can you speak

knowledgeably about fresh *and* frozen seafood sold in your store? If not, who else should we speak with?

Consumers

5. What do consumers look for when shopping for seafood? What about for frozen seafood?

6. Have you noticed different preferences for seafood between groups of customers, such as by age, income, or cultural background? What about for purchases of fresh vs frozen?

7. What are the most popular seafood products (SKUs) you sell? Does this vary by time of year?

Stocking, Marketing, Profitability

8. How many seafood products (SKUs) does your store stock? What percent of SKUs for unprocessed/minimally processed seafood are fresh seafood products, and what percent are frozen seafood? Other types?

9. How important is frozen seafood for overall seafood sales? Does the contribution of frozen seafood

differ by volume of seafood sales vs. profitability of seafood sales?

10. How is inventory managed for fresh seafood? For frozen seafood? Does your store use on-package

labels (sell by, use by, or freeze by) to manage inventory?

11. Who is responsible for placing orders for fresh seafood? For frozen seafood?

12. Who are your main suppliers for fresh seafood? for frozen seafood? How frequently do you receive

deliveries for fresh and frozen seafood?

13. What is your store's advertising and marketing approach for fresh seafood? For frozen seafood?

Seafood Shrink (shrink is a term for seafood loss due to operational issues or theft)

14. What loss or shrink rates are typical for fresh seafood in your store? For Frozen seafood?

What items have the greatest amount of shrink? Least amount of shrink?

15. What is your store's policy for discarding seafood?

16a. Does your store have strategies to reduce seafood loss/shrink? (For example, are products nearing their expiration date cooked for in-store sales at a hot bar? Is ordering adjusted based on historical demand?)

16b. Selling frozen fillets or other minimally processed seafood products with instructions for cooking at home from frozen, instead of thawing prior to cooking, has been proposed as a potential strategy to cut down on wasted seafood in stores and in homes. Do you think that would work in your store? Are there barriers to promoting these types of frozen seafood products to your customers?

17. What opportunities do you see to reduce seafood shrink in your store? What are the biggest barriers for this kind of change?

Is it OK for us to take pictures of seafood offerings in your store?

We would also like to talk to seafood buyers who choose which seafood products to stock in a store or group of stores (e.g., for a chain). Can you share contact information?

Materials S3: Consumer pre- and post-diary survey

PRE-DIARY SURVEY

Please indicate how often you eat seafood that was prepared in each of these places.

	Daily (1)	About twice a week (2)	About once a week (3)	About twice a month (4)	About once a month (5)	A few times a year (6)	Never (7)
Home (your's or someone else's) (1)	○	○	○	○	○	○	○
Sit down restaurant (2)	○	○	○	○	○	○	○
Fast food restaurant or cafeteria (3)	○	○	○	○	○	○	○

What type of product do you **most often** choose when you buy seafood?

○ Canned (1)

○ Cooked (2)

○ Dried (3)

○ Fresh - Fillet (4)

- ◯ Fresh - Whole Fish (5)
- ◯ Frozen - Raw (6)
- ◯ Pickled (7)
- ◯ Processed Frozen (e.g. Fish Sticks) (8)
- ◯ Processed Non-Frozen (e.g. seafood salad) (9)
- ◯ Other (10) ____
- ◯ Don't Know (11)

What are the reasons you prefer this product? Select all that apply.

- ▢ Convenience (1)
- ▢ Habit (2)
- ▢ Know how to prepare it (3)
- ▢ Lasts longer (4)
- ▢ Price (5)
- ▢ Taste (6)
- ▢ Other (7) ____

Please choose the top three most important reasons for choosing seafood in meals compared to other options.

- ▢ Appearance (2)
- ▢ Animal Welfare (3)
- ▢ Convenience (e.g. cooks quickly) (1)
- ▢ Environmental benefits of seafood (4)

- [ ] Habit (5)
- [ ] Health benefits of seafood (6)
- [ ] Price (7)
- [ ] Taste (8)
- [ ] Other (9) ____

Please choose the top three most important reasons why you don't choose seafood more often than you already do.

- [ ] Appearance (2)
- [ ] Animal Welfare (3)
- [ ] Convenience (1)
- [ ] Environmental benefits of other foods (4)
- [ ] Habit (5)
- [ ] Health benefits of other foods (6)
- [ ] Odor (10)
- [ ] Price (7)
- [ ] Seafood spoils more quickly (11)
- [ ] Taste (8)
- [ ] Other (9) ____

Please choose the top three most important factors that affect which seafood ***species*** you choose.

- [ ] Advice from store staff (11)
- [ ] Appearance (2)

☐ Animal Welfare (3)

☐ Convenience (e.g. species is sold pre-seasoned, canned, etc.) (1)

☐ Country of origin (10)

☐ Environment (including sustainability) (4)

☐ Farmed vs. wild (6)

☐ Following a pre-selected recipe (12)

☐ Habit (5)

☐ Price (7)

☐ Taste (8)

☐ Other (9) ____

How many people live in your household?

____

How many children under the age of 18 live in your household all or most of the time?

____

Were you OR either of your parents born outside the US?

○ No (1)

○ Yes, I was born outside the US (2)

○ Yes, my parents were born outside the US (3)

○ Yes, both my parents and I were born outside the US (4)

Display This Question:
If Were you OR either of your parents born outside the US? = Yes, I was born outside the US
Or Were you OR either of your parents born outside the US? = Yes, both my parents and I were born outside the US

Please indicate the country you were born in

____

Display This Question:
If Were you OR either of your parents born outside the US? = Yes, my parents were born outside the

US

Or Were you OR either of your parents born outside the US? = Yes, both my parents and I were born outside the US

Please indicate the country or countries your parents were born in

____

In which state do you currently reside?

▼ Alabama (1) ... I do not reside in the United States (53)

How would you describe the area where you live?

▼ Urban (1) ... Other (4)

Do you live within 15 miles of a coastline, river, or other large body of water?

◯ Yes (23)

◯ No (24)

Compared to the average American, do you think you discard:

◯ More seafood (1)

◯ About the same amount of seafood (2)

◯ Less seafood (3)

To your knowledge, have you ever had a food-borne illness (food poisoning) from seafood?

◯ Yes (23)

◯ No (24)

Some people have specialized experience with seafood. Do any of the following apply to you? Select all that apply.

▢ I commercially fish/harvest seafood (for work) (1)

▢ I fish/harvest recreationally (for fun and/or to get food) (4)

▢ I run or work at a fishing guide company (5)

▢ I sell seafood (at a store, market, etc.) (6)

▢ I work in a restaurant which sells seafood (7)

▢ I work in or study fisheries/marine environments (8)

☐ Other (9) ____

What is your race/ ethnicity? Select all that apply

☐ Asian (11)

☐ Black, non-Hispanic (17)

☐ Hispanic (18)

☐ White, non-Hispanic (19)

☐ Other (21) ____

☐ Prefer not to answer (22)

What is the highest level of education you have completed?

○ Less than high school (11)

○ High school graduate/ GED (12)

○ Some college or Associates' degree (13)

○ Bachelor's degree (14)

○ Graduate school (15)

○ Prefer not to answer (16)

POST-DIARY SURVEY

Thank you for completing our seafood diary survey! We now ask that you complete this short post-survey about your survey experience and a few additional questions about your thoughts and behaviors about seafood. Once you complete this survey, we will process your gift card as a thank you for your participation.

Please enter your three digit participant identifier.

If you need help finding this number or have any other questions, please check out our help page:

____

Please use this space to tell us anything about your experience filling in the diary survey, including challenges you faced, places where the survey might not fully capture what

happened, or ways in which the diary time period was not typical of your normal shopping, cooking or eating.

____

____

Did you learn anything surprising while completing this diary? Please explain.

____

____

The next portion of this survey will ask a few questions about how you decide whether or when to discard seafood and about your seafood purchasing decisions.
How does the seafood package date label affect your view about when seafood is too old to eat? I would avoid eating seafood __________.

- ◯ Some time before the date on the package (1)
- ◯ On the date on the package (2)
- ◯ After the date on the package (3)
- ◯ Other (4) ____
- ◯ The date label is not a major factor in my decisions (5)

Display This Question:
If How does the seafood package date label affect your view about when seafood is too old to eat? I... = Some time before the date on the package

How many days before the date label on the package do you usually throw away seafood?

____

Display This Question:
If How does the seafood package date label affect your view about when seafood is too old to eat? I... = After the date on the package

How many days after the date label on the package do you usually throw away seafood?

____

What is the ***longest*** amount of time in days that you would keep ***fresh*** seafood in your fridge before preparing it to eat?

____

What is the ***longest*** amount of time in days that you would keep ***frozen*** seafood in your freezer before preparing it to eat?

____

What is the ***longest*** amount of time in days you would keep ***canned*** seafood before preparing it

to eat?

____

Please use the slider to select how many hours it is safe to leave **cooked** seafood at room temperature. You may select half hours by moving the slider in between whole numbers.

Select 0 if you feel that it is not safe to leave cooked seafood at room temperature for any amount of time.

	Cooked seafood can be left at room temperature for:<br>0 1 2 3 4 5 6 7 8 9 10 11 12
Hours ()	

How do you decide when seafood may no longer be good to eat? Check all that apply

- ☐ Amount of time left out of refrigerator (1)
- ☐ Appearance (2)
- ☐ Date label (3)
- ☐ Odor (4)
- ☐ Time since purchase (5)
- ☐ Time since cooking (6)
- ☐ Other (7) ____________________

Carry Forward Selected Choices from "How do you decide when seafood may no longer be good to eat? Check all that apply"

Please rank the factors that you use to decide if seafood is no longer good to eat (1 being most important)

To rank the listed items, drag and drop each item below.

______ Amount of time left out of refrigerator (1)
______ Appearance (2)
______ Date label (3)
______ Odor (4)

______ Time since purchase (5)
______ Time since cooking (6)
______ Other (7)

For the previous questions about factors that affect when you decide seafood is no longer good to eat, do your answers differ based on the type of seafood?

- ○ Yes (1)
- ○ No (2)

Display This Question:
If For the previous questions about factors that affect when you decide seafood is no longer good to... = Yes

Please explain how the type of seafood affects when you decide that it is no longer good to eat

____

____

Nutritional advice often tells us to eat more seafood. How does this advice affect the amount of seafood your household buys?

- ○ No effect (1)
- ○ Buy a little more than we would otherwise (2)
- ○ Buy a lot more than we would otherwise (3)
- ○ Other (4) ____

Display This Question:
If Nutritional advice often tells us to eat more seafood. How does this advice affect the amount of... = Buy a little more than we would otherwise
Or Nutritional advice often tells us to eat more seafood. How does this advice affect the amount of... = Buy a lot more than we would otherwise

How often does this extra seafood that your household buys tend to go bad before you can eat it?

- ○ Never (1)
- ○ Sometimes (2)
- ○ About half the time (3)
- ○ Most of the time (4)

- Always (5)

To your knowledge, how often was seafood that was labeled as fresh in stores previously frozen?

- Never (1)
- Sometimes (2)
- Often (3)
- Always (4)

For the following, please rank your level of knowledge for each listed skill from not at all knowledgeable to extremely knowledgeable.

	Not knowledgeable at all (1)	Slightly knowledgeable (2)	Moderately knowledgeable (3)	Very knowledgeable (4)	Extremely knowledgeable (5)
How to assess seafood quality (1)	○	○	○	○	○
How to assess seafood safety (including spoilage) (2)	○	○	○	○	○
How to cook seafood (3)	○	○	○	○	○
How to store seafood (4)	○	○	○	○	○
How to find out where seafood comes from (5)	○	○	○	○	○

The meaning of environm'l or sust. labels on seafood (6)	○	○	○	○	○

Where do you prefer to get information about seafood including which types to buy and cook? Check all that apply.

- ☐ Blogs (1)
- ☐ Social media – friend (2)
- ☐ Social media - brand (3)
- ☐ Online news websites (4)
- ☐ Magazines (5)
- ☐ Television (6)
- ☐ Radio (7)
- ☐ Print newspapers (8)
- ☐ Friends and family (9)
- ☐ I don't seek out this information (10)
- ☐ Other (11) ____

The final portion of this survey will ask about your attitudes towards cooking and handling seafood in your household.

The members of my household agree on when it is time to discard seafood _________.

- ○ Never (1)
- ○ Rarely (2)

○ Sometimes (3)

○ Often (4)

Compared to the average American, how much seafood do you throw away?

○ More seafood (1)

○ About the same amount of seafood (2)

○ Less seafood (3)

Compared to the average American, do you throw away:

○ More *food* (1)

○ About the same amount of *food* (2)

○ Less *food* (3)

How important are each of the following factors in motivating you to reduce the amount of seafood your household throws away?

	Not at all important (1)	Slightly important (2)	Moderately important (3)	Very important (4)	Not Applicable (5)
Thinking about people without enough to eat (1)	○	○	○	○	○
Thinking about saving money (2)	○	○	○	○	○
Feeling regret about time spent shopping, storing, or preparing food not eaten (3)	○	○	○	○	○
Wanting to manage my home efficiently (4)	○	○	○	○	○
Feeling guilty about waste in general (5)	○	○	○	○	○
Thinking about the greenhouse gases, energy and water resources it took to get the food to my plate	○	○	○	○	○

(6)					
Thinking about the fish/shellfish that died (7)	◯	◯	◯	◯	◯
Thinking about the idea that I can make a difference through my actions (8)	◯	◯	◯	◯	◯
Wanting to set an example for my children (9)	◯	◯	◯	◯	◯
Other (please specify) (10)	◯	◯	◯	◯	◯

How much effort do you currently make to minimize the amount of seafood you throw away?

◯ None (1)

◯ A little (2)

◯ A medium amount (3)

◯ A lot (4)

Some chefs are proposing the idea of buying frozen seafood and cooking it without defrosting it. Have you ever tried this?

◯ Yes (1)

◯ No (2)

Display This Question:
If Some chefs are proposing the idea of buying frozen seafood and cooking it without defrosting it.... = Yes

When you cook seafood directly from frozen without defrosting it first, do you notice a difference in flavor from seafood that is defrosted?

◯ Yes (1)

◯ No (2)

Display This Question:
If Some chefs are proposing the idea of buying frozen seafood and cooking it without defrosting it.... = No

Would you be willing to try cooking seafood directly from frozen without defrosting it first?

◯ Yes (1)

◯ No (2)

Display This Question:

If Some chefs are proposing the idea of buying frozen seafood and cooking it without defrosting it.... = No

Why or why not?

____

Below is a recipe, developed by chefs, for cooking seafood direct from frozen. Please take a look and respond to the two statements about your feelings about the recipe.

Roasted Tilapia with Creamy Tartar Sauce Serves: 4 - 2 tablespoons olive oil - 4 frozen tilapia fillets - 1-pound red potatoes, halved - 1 bunch asparagus, ends trimmed - 1 lemon, juiced - 2 teaspoons old bay seasoning - 1 cup plain Greek yogurt - ½ cup pickle relish - Salt and pepper to taste

1. Preheat oven to 350 degrees.
2. On a baking sheet tray, drizzle olive oil. Place tilapia on one section of the tray, potatoes on another section and asparagus in remaining section. Season with lemon juice, old bay, salt and pepper.
3. Place sheet tray in oven and roast for about 30 minutes, or until fish is cooked through and potatoes are tender.
4. In a small bowl, mix together yogurt and pickle relish. Season with salt and pepper to taste 5. Serve tilapia, potatoes and asparagus with Greek yogurt tartar sauce.

How do you think this recipe would taste?

◯ Extremely good (1)

◯ Somewhat good (2)

◯ Neither good nor bad (3)

◯ Somewhat bad (4)

◯ Extremely bad (5)

Would you try preparing this recipe?

◯ Definitely not (1)

◯ Probably not (2)

◯ Maybe (3)

○ Probably would (4)

○ Definitely would (5)

What information or tools, if any, would be helpful in reducing the amount of seafood your household throws out? Check all that apply

☐ How to store seafood (1)

☐ How long seafood can be left out unrefrigerated (2)

☐ Freezing seafood products (3)

☐ How to interpret food date labels (e.g., USE by, BEST if used by, etc.) (4)

☐ How to assess whether seafood may have become unsafe (5)

☐ How to buy and cook with seafood that is frozen (6)

☐ Apps to help with shopping or portion planning (7)

☐ Smaller seafood package sizes (8)

☐ Other (please specify) (9) ____

☐ I don't need anything (10)

Thank you for completing the post survey for our seafood diary.

Materials S4: Consumer seafood diary questionnaire

In the preparatory materials, respondents were urged to shop, prepare food and discard food as "normally" as possible during the diary period. They will also be requested to take a photo of the packaging (or save it) so they will have information about the date labels.

1. Please enter your three digit participant identifier:
2. What date is this diary entry for? ________

EVERYONE GETS THESE 3 QUESTIONS

A. Did you or a household member buy seafood yesterday, NOT at a restaurant?

Yes; No

B. Did you or a household member prepare seafood yesterday?

Yes; No

C. Did you or a household member throw out or compost seafood yesterday?

Yes; No

For those who answered yes to any of these questions, there will be followup series of questions

If yes to A:

Answer the following questions for the first seafood item that was bought yesterday, NOT from a restaurant [e.g., package of shrimp]. If there were multiple seafood items bought yesterday, you will have the chance to answer the following questions about each one separately. The order does not matter.

- Where was it purchased? supermarket; fish store or dock; don't know; other [text]
- What kind of seafood was bought? (options: shrimp, salmon, catfish, tuna canned, tuna fresh, tilapia, pollock, cod, pangasius, crabs, clams, other or mixed (textbox), don't know)
- How much of this item was bought, in servings? One serving is about 3 oz, or the size of a deck of cards. If the dish includes other foods besides seafood, try to estimate just how many servings of seafood were in it. Please give your best guess of the number of seafood servings.[text]
- What form was the item in? (options: fresh-fillet, fresh-whole fish, frozen, canned, cooked, processed frozen (e.g., fish sticks), processed nonfrozen (e.g., seafood salad), dried, pickled, other, don't know)
- If there was a date on the package, what did it say? Please include both text and date, e.g., "use by January 15." [Text OR Don't know OR No date]
- Any more seafood items to enter? Yes; No [If yes, restart questions]

If yes to B:

- Please answer the following questions for the first seafood item that was prepared yesterday [e.g., package of shrimp]. If there were multiple seafood items, you will have the chance to answer the following questions about each one separately. The order does not matter.
- Was this the seafood item that was prepared?: [list items purchased including date– click one or add new]?

If added new, ask:

- Where was it purchased? supermarket; restaurant; fish store or dock; don't know; other [text]

If restaurant:

- Any more seafood items to enter? Yes; No [If y, restart questions]

If anything other than restaurant:

- What kind of seafood was prepared? (options: shrimp, salmon, catfish, tuna canned,

tuna fresh, tilapia, pollock, cod, pangasius, crabs, clams, other or mixed (textbox), don't know)

- What form was the item in before it was prepared? (options: fresh-fillet, fresh-whole fish, frozen, canned, cooked, processed frozen (e.g., fish sticks), processed nonfrozen (e.g., seafood salad), dried, pickled, other, don't know)
- If there was a date on the package, what did it say? Please include both text and date, e.g., "use by January 15." [Text OR Don't know OR No date]
- How much of this seafood was prepared, in servings? One serving is about 3 oz, or the size of a deck of cards. If the dish includes other foods besides seafood, try to estimate just how many servings of seafood were in it. Please give your best guess of the number of seafood servings. ____ [text]
- How was the seafood prepared? Put name of dish or brief description [Text.]

If seafood was frozen:

- Was the frozen seafood defrosted before preparing it, or cooked direct from frozen?

All continue here:

- Any more seafood items to enter? Yes, No [If y, restart questions]

If yes to C:

- Please answer the following questions for the first seafood item that was discarded or composted yesterday [e.g., package of shrimp]. If there were multiple seafood items, you will have the chance to answer the following questions about each one separately. The order does not matter.
- Was this the seafood item that was discarded or composted?: [list items purchased or prepared including date – click one or add new]?

If added new, ask:

- Where was it purchased? supermarket; restaurant; fish store or dock; other [text]

If restaurant:

- Any more seafood items to enter? Yes; No [If y, restart questions]

If not restaurant:

- What kind of seafood was discarded or composted? (options: shrimp, salmon, catfish, tuna canned, tuna fresh, tilapia, pollock, cod, pangasius, crabs, clams, other or mixed (textbox), don't know)
- What form was the item in? (options: fresh-fillet, fresh-whole fish, frozen, canned, cooked, processed frozen (e.g., fish sticks), processed nonfrozen (e.g., seafood salad), dried, pickled, other, don't know)
- If there was a date on the package, what did it say? Please include both text and date, e.g., "use by January 15." [Text OR Don't know OR No date]
- How much of this item was thrown out or composted, in servings? One serving is about 3 oz, or the size of a deck of cards. If the dish includes other foods besides seafood, try to

estimate just how many servings of seafood were in it. Please give your best guess of the number of seafood servings. [text]

- Please give your best guess about the # of servings of seafood. [text]
- Had the seafood been previously prepared, e.g., cooked or put into a seafood salad? Yes; No
- What were reasons for throwing it out or composting it? [check all that apply]
    - (too long since purchased; smelled; looked bad; texture was "off", past date; the seafood dish wasn't good (burned, bad recipe, etc); the seafood itself did not taste right; forgot; made or bought too much; other person felt it should be discarded; couldn't finish and didn't eat leftovers; don't know; other [text])
  - *If "yes" to "looked bad"* – What specifically looked bad about the item?
    - Cloudy eyeball
    - Color change
    - Looked dry
    - Looked slimy
    - Had freezer burn
    - Moldy
    - Other __________
    - Don't know
  - *If "yes" to "made too much"* – Why did you make too much?
    - Wanted to make sure there was enough food for everyone eating
    - Bought too much of the item, and prepared all of it
    - Like to prepare enough for leftovers, but my I (or my family) did not eat the leftovers or reuse them in another meal
    - Other ______
- Would you like to share a photo of this seafood? Please do not include anyone's face in the photo. [option to upload]
- Any more seafood items to enter? Yes, No [If y, restart questions]

Materials S5: Consumer focus group guide

1. First name, where you're from, favorite seafood dish.
2. Think about going to the store to buy seafood. Imagine looking across the array of fresh and frozen seafood available in the fresh and frozen sections. On this paper, jot down three (or more) of the most important things you think about when choosing which specific seafood item to buy?
    a. Moderator: Write on board (note how many select each item).
    b. Anything missing here? (e.g., taste, species, price, freshness, appearance, where it comes from, etc.)
    c. Allow discussion of what they value in these qualities. But cut it off if it goes on too long.
3. How do you decide whether to buy seafood fresh or frozen?
    a. Which is healthier, tastes better, costs less, is more convenient, is wasted less – fresh or frozen? (eyes closed show of hands for each of these. Moderator record #including option for DK.)

After each question, ask for a few people to elaborate on those choices if they'd like

b. How often do you buy frozen seafood?
c. Eyes closed show of hands: True or false: most "fresh" seafood you see in the store was originally frozen and then defrosted for sale. (moderator record # including DK)
    i. For those who didn't know- what do you think about this?
    ii. For those that did, how did you find out? Did it affect your purchasing decisions?

4. What do you think of the idea of cooking seafood direct from frozen, that is, not defrosting it first?
    a. These are recipes (distribute 2) that were created by chefs to give people a way to cook direct from frozen. How do they look to you? Would you make them?
5. How long do you keep fresh seafood in your refrigerator (prepared or otherwise)?
    a. Prompts:
        i. How do you know if it's been too long?
        ii. Who decides?
6. How long do you or would you keep frozen seafood in your freezer (prepared or otherwise)?
    a. Prompts:
        i. How do you know if it's been too long?
        ii. Who decides?
7. How often do you end up throwing away seafood because you couldn't make or eat it in time?
8. Some researchers are exploring the idea of encouraging consumers to buy seafood frozen and to cook directly from frozen, as a way to reduce waste and the lost money that goes with that.
    a. What do you think of this idea?
    b. What are some ways to convince customers this is a good idea?
    c. What do you think are the top reasons this strategy might NOT succeed?
    d. We've talked about a lot of issues related to this idea. What do you think is most important for the team to be thinking about?

Have we missed anything the researchers should be thinking about?

Ask participants to fill out a brief demographic questionnaire. This is a half-page form asking for ID#, age ___, race/ethnicity (give categories), education level (categories), income (categories), gender ___, zip code.
Tell them that if they are interested to receive an email of the study report, they can leave their email address. (Bring 3x5 cards for this.)

Materials S6: Qualitative Codebook

Code Group	Code	Atlas Code	Code Description	*Examples*
PARTICIPANT CHARACTERISTICS	age	participant - age	Participant refers to their age	
	cooking experience	participant - cooking experience	Cooking experience/ opinions	

	family culture	participant - family culture	Recollection or knowledge from childhood/ family upbringing; discusses cultural factors in decision	
	family size	participant - family size	Participant refers to their family size	
ACTION	buy	action - buy	Discusses buying behaviors	
*usually secondary indicate what action the participant associates a particular term or behavior with	cook from frozen	action - cook from frozen	Use for all general comments about cook from frozen (NOT for recipe feedback)	
	prep	action - prep	Discusses preparation & cooking behaviors; use for ANY consumer handling of a product that is not related to freezing, thawing, or cook from frozen	
	recipe feedback	action - recipe feedback	Use for all comments related to the cook from frozen recipes	
	storage	action - storage	Discuss storage behaviors; discuss how long they store a product; discuss storage time as a factor in decisions	
	thaw	action - thaw	Thawing practices or opinions	
	wash	action - wash	Participant discusses washing a product	
	waste	action - waste	Discuss waste behaviors	
CONSIDERATIONS	convenience & time	considerations - convenience & time	Convenience/ ease of a product, amount of time required for a product	*"can quickly make a meal"*<br>*"so easy to cook"*<br>*"fresh more time consuming"*<br>*"too much prep work"*
Factors, not covered in other categories, that affect decisions	cooks well	considerations - cooks well	How well the product cooks	
	fresh frozen compare	considerations - fresh frozen compare	Participant directly compares fresh and frozen	
	mood	considerations - mood	Participants mood plays a role in their behavior	*"Impulse buy"*<br>*"What I feel like eating"*
	outside opinion	considerations - outside opinion	Participant seeks outside opinion for a decision; this includes other family members	*"Employee recommendation"*<br>*"Second opinion"*<br>*"News coverage"*
	plan/ recipe	considerations - plan/ recipe	Have a plan/ recipe/ purpose for the item; discuss following a recipe or cookbook	*Meal planning*<br>*"Frozen better for estimating amount"*

	routine/ special	considerations - routine/ special	Participant discusses decision related to routine or special uses	*"I'll buy fresh for a special occasion"* *"Frozen is good for everyday"* *"splurge"*
	versatility	considerations - versatility	Versatility of a product	*"Have many preparation options"*
	other	considerations - other	A factor that affects decisions that isn't listed	
COST	value	cost - value	The value of a product is mentioned or tactics for getting a good value/ lower price are discussed; sales are mentioned	*"Price per piece"* *"Cheaper in bulk"* *"On sale"* *"Coupon"*
	other	cost - other	General discussion of cost or cost discussion that doesn't include value codes	
FEELINGS/ EMOTIONS	confident	emotion - confident	Participant indicates familiarity/ confidence with an item, method, or concept	*"know what fresh should look like"* *"previous experience with poaching"* *"intuition"*
	guilt	emotion - guilt	Participant indicates guilt	
	misconception	emotion - misconception	Used for misconceptions stated by participant; code if coder is unsure that a statement is true	
	recommendation	emotion - recommendation	Participant provides a direct recommendation to researchers	
	revulsion	emotion - revulsion	Participant indicates revulsion/ disgust; can be mild	
	seafood exception	emotion - seafood exception	Participant indicates that seafood is unique/ an exception, when compared to other food products	*"seafood goes bad faster"*
	stubborn	emotion - stubborn	Participant says they're stubborn or set in their ways	
	try it	emotion - try it	Participant says they would be willing to try something that they haven't or are curious about something	*"give it a shot"* *"willing to try something new"* *"need to learn how"* *curiosity*
	unsure	emotion - unsure	Participant is unsure of, confused by, or unfamiliar with something	*"how to determine freshness"* *"doubt"* *"never poached before"* *"not used to the idea"*
	other	emotion - other		

FREEZE	freezer damage	freeze - freezer damage	Damage caused to product by freezing	*"Freezer burn"<br>"Freezing causes fish to get soupy"*
	re-freeze	freeze - re-freeze	Anything related to re-freezing a thawed product	*"Re-freezing affects integrity"<br>"Don't want to freeze-thaw"*
FROM FROZEN WILLINGNESS<br>Based on ranking question	1 not at all willing	from frozen willingness - 1 not at all willing	Based on ranking question	
	2 slightly willing	from frozen willingness - 2 slightly willing	Based on ranking question	
	3 moderately willing	from frozen willingness - 3 moderately willing	Based on ranking question	
	4 very willing	from frozen willingness - 4 very willing	Based on ranking question	
	5 extremely willing	from frozen willingness - 5 extremely willing	Based on ranking question	
FROZEN FREQUENCY	1 - never	purchase frozen frequency - 1 - never	Based on ranking question	
	2 - rarely	purchase frozen frequency - 2 - rarely	Based on ranking question	
	3 - sometimes	purchase frozen frequency - 3 - sometimes	Based on ranking question	
	4 - often	purchase frozen frequency - 4 - often	Based on ranking question	
	5 - always	purchase frozen frequency - 5 - always	Based on ranking question	
ISSUES	cooking issues	issues - cooking issues	Problems that arise during cooking	*"Overcook in oven"<br>"Want to fill pan"<br>"Uneven cooking"*
Issues: *General issues that arise in the storage and preparation of a product	food safety	issues - food safety	Safety of a product, including fully cooking and contamination are mentioned; additional things added to products that concern consumers; safety regulations	*"Refreeze has bacteria"<br>"Worry about contamination"<br>Power outage concerns<br>Methods for checking doneness<br>Additives, Mercury, antibiotics<br>"USDA ensures safety"*
	leftovers	issues - leftovers	Leftover issues, practices, opinions, amount	
	other	issues - other	General issues that arise in the storage and preparation of a product	
LOCATION	asian market	location - asian market		
Specific location that participant mentions they buy seafood	boat/ dock	location - boat/ dock		
	seafood counter/ market	location - seafood counter/ market	Independent seafood market	
	supermarket - chain	location - supermarket - chain	Large chain store	*Walmart<br>Target<br>Safeway*

	supermarket - special	location - supermarket - special	Chain that is considered specialty/ natural/ organic	*Whole Foods<br>Trader Joe's<br>MOM's*
	wholesaler	location - wholesaler		
	other	location - other	Specific location where a participant buys seafood, not otherwise coded	
PRODUCT	fresh	product - fresh	Fresh or freshness	
	bought frozen	product - frozen	Product is purchased frozen by the consumer	
	frozen at home	product - home frozen	Product is frozen at home by consumer	
	live	product - live		
	other product type	product - other	Not fresh, frozen, live	
	previously frozen	product - previously frozen	Product was frozen by store or processor by thawed prior to sale	
	species	product - species	Participant mentions a specific species of seafood	
PRODUCT ATTRIBUTES	appearance	attributes - appearance	Appearance of product discussed; participant says something about look of a product	*"Moist"<br>"Look"<br>"Look at all sides"*
	availability/ seasonality	attributes - availability/ seasonality	Participant mentions availability of product or seasonality	*"Easier to find frozen"<br>"Time of year affects decision"*
	brand	attributes - brand	Brand of product	
	color	attributes - color	Color of product	
	health	attributes - health	Participant discuss health/ medicinal/ nutritional aspects	*"Fresh is healthier"<br>"Frozen loses nutrients"*
	individually wrapped	attributes - individually wrapped	Reference to individually packaged product	
	non-meat parts	attributes - non-meat parts	Parts of seafood beside the meat are discussed	*Skin, juices, scales, shells bones*
	package	attributes - package	Anything related to external packaging of product	*Cooking directions on label<br>Date label<br>Label/ package design<br>Shrink wrapped*
	prepped in store	attributes - prepped in store	Product is cooked/ prepared in store OR restaurant	*"fat cut"<br>"cooked/ steamed"<br>"clean fish in front of you"<br>"pre-seasoned"*

	production	attributes - production	Factors of production process are discussed; anything that occurs before reaching the store; including country of origin	*"Fish eat waste"<br>GMOs<br>Wild v. farmed<br>Labor conditions<br>Organic/ sustainable<br>"Frozen immediately"*
	quality	attributes - quality	Participant discusses general quality, uses good/ bad	*"Not good when bought"*
	quantity	attributes - quantity	Amount of product in package	*"Can prep frozen in smaller quantities"<br>"You get more frozen product per bag"*
	size	attributes - size	Size of the actual product	*"The shrimp are huge"<br>Thickness of fish fillet*
	smell	attributes - smell	Product smell	
	taste	attributes - taste	Product taste	*"Family doesn't like it"*
	texture	attributes - texture	Texture/ integrity of product	*"Want to touch the product"<br>"Slimy"<br>"Fresh dries out"<br>"Refreezing harms integrity"<br>"Frozen gets soupy"*
	water content	attributes - water content	Water content/ excess water mentioned	
	other	attributes - other	A factor about the product not otherwise listed	
STORE ATTRIBUTES	clean	store - clean	Cleanliness of store or seafood counter	
	close	store - close	Proximity to store	
	coast/ local	store - coast	Store is close to coast or seafood is local	
	hours	store - hours	Store hours	
	marketing	store - marketing	Store marketing or presentation of seafood	
	no preference	store - no preference	Participant indicates no preference for a single store or chain	
	reputation/ trust	store - reputation/ trust	Reputation or trust of a store; also prefer independent chain	*"reputation"<br>"false advertising"<br>"misleading"<br>"more independent stores"<br>"not chains"*
	selection	store - selection	Selection or variety of seafood in the store	*"not much variety"<br>"rare species"*
	usual	store - usual	Participant indicates a single location that they usually go to	

	other	store - other	Other factors that affect decision of where to shop	
WASTE<br><br>*General preparation comments get coded as "action - waste"	animals	waste - animals	Give leftover food to animals	*"Feed to cat"*
	donate	waste - donate	Give leftover food away	*"Give to homeless"*<br>*"Give to neighbor"*
	don't waste	waste - don't waste	Participant says they do not waste seafood or waste very little	*"I use every bit"*<br>*"Process into another dish"*<br>*"Kids eat everything"*
	forget	waste - forget	Forget a product and end up wasting it	*"Lost in fridge/ freezer"*
	gone bad	waste - gone bad	Participant specifically discusses a product going bad/ what makes a product bad	
	life	waste - life	Some life event interferes with using a product	*"Things come up"*<br>*"Went on vacation"*
	waste aversion	waste - waste aversion	General strategies to avoid wasting food	

Materials S7: Recipe used in survey (developed by Drexel Food Lab)

Roasted Tilapia with Creamy Tartar Sauce Serves: 4 - 2 tablespoons olive oil - 4 frozen tilapia fillets - 1-pound red potatoes, halved - 1 bunch asparagus, ends trimmed - 1 lemon, juiced - 2 teaspoons old bay seasoning - 1 cup plain Greek yogurt - ½ cup pickle relish - Salt and pepper to taste

1. Preheat oven to 350 degrees.
2. On a baking sheet tray, drizzle olive oil. Place tilapia on one section of the tray, potatoes on another section and asparagus in remaining section. Season with lemon juice, old bay, salt and pepper.
3. Place sheet tray in oven and roast for about 30 minutes, or until fish is cooked through and potatoes are tender.
4. In a small bowl, mix together yogurt and pickle relish. Season with salt and pepper to taste 5. Serve tilapia, potatoes and asparagus with Greek yogurt tartar sauce.

Materials S8: Recipes used in focus group (developed by Drexel Food Lab)

Butter Poached Garlic Shrimp

Serves: 6

- 1/2 cup butter
- 1/2 cup water
- 10-15 garlic cloves, chopped
- 1 (2 pound) bag Peeled and Deveined Frozen Shrimp
- 1 bunch scallions, chopped
- salt and pepper to taste

1. In a medium pot over medium heat add butter, water and garlic. Season with salt and pepper. Bring to a simmer and melt butter.
2. Add shrimp and cook for about 15 minutes, or until shrimp turn pink. Season with salt, pepper, and scallions.
3. Serve with grilled bread or over pasta.

Roasted Tilapia Sheet Tray Dinner with Yogurt Tartar Sauce

Serves: 4

- 2 tablespoons olive oil
- 4 frozen tilapia fillets
- 1-pound red bliss potatoes, halved
- 1 bunch asparagus, ends trimmed
- 1 lemon, juiced
- 2 teaspoons old bay seasoning
- 1 cup plain Greek yogurt
- ó cup pickle relish
- Salt and pepper to taste

1. Preheat oven to 350 degrees.
2. On a baking sheet tray, drizzle olive oil. Place tilapia on one section of the tray, potatoes on another

section and asparagus in remaining section. Season with lemon juice, old bay, salt and pepper.
3. Place sheet tray in oven and roast for about 30 minutes, or until fish is cooked through and potatoes
are tender.
4. In a small bowl, mix together yogurt and pickle relish. Season with salt and pepper to taste
5. Serve tilapia, potatoes and asparagus with Greek yogurt tartar sauce.

Table S1: Demographics for diary completers and full consumer survey sample

Demographic variable	Screener (n=282)	Pre-Diary Survey (n=121)	Diary (n=43)	US Population
Age				
18-29	25	22	21	21
30-44	41	53	49	25
45-64	28	20	26	33
65+	6	5	5	20
Prefer not to answer	1	0	0	0
Gender - % Female	73	72	79	51
Annual Household Income				
< $24,999	21	8	5	20
$25,000 - $49,999	20	15	26	22
$50,000 - $74,999	23	39	19	17
$75,000 - $125,000	24	37	40	27*
> $125,000	9	10	12	15**
Prefer not to answer	3	0	0	0
Restaurant/grocery experience	33	18	14	NA
Household size: Mean (SD)		3.3 (1.5)	2.8 (1.2)	2.54
# Children < 18 yrs: Mean (SD)		1.5 (2.8)	0.9 (1.0)	
Education				
Less than high school		2	0	10
High school/GED		8	7	29
Some college		22	21	27
BA		47	49	21
Graduate school		21	23	13
Geography (self-characterized)				
Urban		60	53	
Suburban		35	40	

Rural	3	5	
Other	2	2	
Race/Ethnicity			
Asian	7	14	6
Black, non-Hispanic	16	11	13
White, non-Hispanic	70	70	61
Hispanic	4	0	18
Other	2	2	2
Prefer not to answer	1	2	0
Live within 15 mi of coastline, river, other large body of water	64	70	
Born outside US or parents	19	19	

Notes: # respondents to each question vary slightly. *$100,000-149,999; **>$150,000

Table S2: Relationship between "4 P's" categories of seafood waste drivers and 11 categories of overall food waste drivers identified by National Academies of Sciences & Medicine Committee on a Systems Approach to Reducing Consumer Food Waste*

	Proficiency	Perception/ Knowledge	Planning/ Convenience	Perishability
A. Consumers' knowledge, skills, and tools				
B. Consumers' capacity to assess risks associated with food waste				
C. Consumers' goals with respect to food and nutrition				
D. Consumers' recognition and monitoring of their food waste				
E. Consumers' psychological distance from food production and disposal				
F. Heterogeneity of consumers' food preferences and diets				
G. The convenience or inconvenience of reducing food waste as part of daily activities;				
H. Marketing practices and tactics that shape consumers' food behaviors				
I. Psychosocial and identity-related norms related to food consumption and waste				

J. Factors in the built environment (including in household and retail environments) and the food supply chain; and<br>K. Policies and regulations at all levels of government.				

* Committee on a Systems Approach to Reducing Consumer Food Waste; Board on Environmental Change and Society; Food and Nutrition Board; Division of Behavioral and Social Sciences and Education; Health and Medicine Division; National Academies of Sciences, Engineering, and Medicine *A National Strategy to Reduce Food Waste at the Consumer Level*; Schneeman, B.O., Oria, M., Eds.; National Academies Press: Washington, D.C., 2020; p. 25876; ISBN 978-0-309-68073-8.
